# Emerging Therapeutic Strategies in Prostate Cancer: Targeted Approaches Using PARP Inhibition, PSMA-Directed Therapy, and Androgen Receptor Blockade with Olaparib, Lutetium (^177^Lu)Vipivotide Tetraxetan, and Abiraterone

**DOI:** 10.3390/jcm15020685

**Published:** 2026-01-14

**Authors:** Piotr Kawczak, Tomasz Bączek

**Affiliations:** 1 Department of Pharmaceutical Chemistry, Faculty of Pharmacy, Medical University of Gdańsk, 80-416 Gdańsk, Poland; tomasz.baczek@gumed.edu.pl; 2 Department of Nursing and Medical Rescue, Institute of Health Sciences, Pomeranian University in Słupsk, 76-200 Słupsk, Poland

**Keywords:** prostate cancer, PARP inhibitor, olaparib, PSMA-targeted therapy, lutetium Lu 177 vipivotide tetraxetan, abiraterone, androgen receptor blockade, mCRPC, precision oncology, targeted therapy

## Abstract

Prostate cancer is one of the most common malignancies in men, and advanced or metastatic disease remains associated with substantial morbidity and mortality. Therapeutic progress in recent years has been driven by the introduction of targeted treatment strategies, notably poly (ADP-ribose) polymerase (PARP) inhibitors, prostate-specific membrane antigen (PSMA)–directed radioligand therapy (RLT), and androgen receptor pathway inhibitors (ARPIs). This review summarizes evidence from phase II and III clinical trials, meta-analyses, and real-world studies evaluating the efficacy, safety, and clinical integration of olaparib, lutetium (^177^Lu) vipivotide tetraxetan, and abiraterone in advanced prostate cancer. Emphasis is placed on the practical clinical application of these agents, including patient selection, treatment sequencing, and combination strategies. PARP inhibition with olaparib has demonstrated clear benefits in metastatic castration-resistant prostate cancer (mCRPC) with homologous recombination repair (HRR) mutations, particularly BRCA1/2 alterations. PSMA-directed RLT offers a survival advantage in PSMA-positive mCRPC following AR pathway inhibition, with distinct toxicity considerations that influence patient selection. Abiraterone remains a cornerstone therapy across disease stages and plays an important role both as monotherapy and as a combination partner. Emerging data suggest a potential synergy between PARP inhibitors and AR-targeted agents, while also highlighting the limitations of biomarker-unselected approaches. We conclude that the optimal use of PARP inhibitors, PSMA-targeted RLT, and ARPIs requires a personalized strategy guided by molecular profiling, functional imaging, prior treatment exposure, and safety considerations. This clinically focused overview aims to support evidence-based decision-making in an increasingly complex treatment landscape.

## 1. Introduction

Prostate cancer is the second most frequently diagnosed malignancy in men and remains a leading cause of cancer-related mortality worldwide [1,2,3]. While localized disease is often effectively managed with surgery or radiotherapy, advanced and metastatic prostate cancer continue to pose substantial therapeutic challenges. Disease progression typically follows a trajectory from hormone-sensitive prostate cancer to metastatic castration-resistant prostate cancer (mCRPC), a state characterized by persistent androgen receptor (AR) signaling despite castrate levels of testosterone. This transition is driven by a spectrum of molecular alterations, including AR amplification, intratumoral androgen synthesis, defects in DNA damage repair (DDR), and overexpression of prostate-specific membrane antigen (PSMA) [4,5,6,7].

Over the past decade, the therapeutic landscape of advanced prostate cancer has evolved rapidly, driven by the clinical translation of molecularly targeted strategies. Among these, three therapeutic pillars have emerged as central to contemporary management: (1) poly(ADP-ribose) polymerase (PARP) inhibition, particularly with olaparib, targeting tumors with homologous recombination repair (HRR) deficiencies; (2) PSMA-targeted radioligand therapy (RLT), most notably lutetium (^177^Lu) vipivotide tetraxetan (^177^Lu-PSMA-617), which enables targeted systemic radiation delivery; and (3) intensified AR pathway inhibition with agents such as abiraterone acetate, a CYP17A1 inhibitor that suppresses extragonadal androgen synthesis and enhances androgen deprivation therapy (ADT)–mediated AR blockade [8,9,10,11]. While these approaches are often discussed separately, their increasing convergence in clinical practice underscores the need for an integrative framework that considers biomarkers, disease biology, and treatment sequencing.

PARP inhibitors have fundamentally reshaped the treatment of HRR-deficient mCRPC. The pivotal PROfound trial demonstrated that olaparib significantly improved radiographic progression-free survival (rPFS) and overall survival (OS) in patients with BRCA1/2 and other HRR gene alterations following progression on AR signaling inhibitors (ARSIs) [12,13,14]. Subsequent analyses and real-world evidence reinforced the clinical importance of genomic profiling and supported regulatory approvals of olaparib for HRR-mutated mCRPC across multiple jurisdictions [15,16,17]. More recently, combination strategies such as olaparib plus abiraterone have been evaluated in first-line mCRPC. Trials including PROpel and MAGNITUDE have suggested potential activity beyond BRCA-mutated disease, while also highlighting important biological limitations and heterogeneity of benefit, particularly in biomarker-unselected populations [18,19,20].

SMA-targeted RLT represents another major advance, leveraging the high and relatively selective expression of PSMA in advanced prostate cancer. The phase III VISION trial established that ^177^Lu-PSMA-617, when added to standard of care, significantly improved OS and rPFS in patients with PSMA-positive mCRPC previously treated with ARSIs and taxane chemotherapy [21]. Earlier phase II data from the TheraP trial demonstrated superior prostate-specific antigen (PSA) response rates and a more favorable toxicity profile compared with cabazitaxel [22,23]. Subsequent real-world and registry-based studies have confirmed the reproducibility of these outcomes, supporting the incorporation of PSMA-RLT into clinical guidelines and routine practice [24,25].

AR pathway inhibition remains a cornerstone of therapy across the disease continuum. Abiraterone acetate, administered with prednisone, demonstrated significant OS benefits in both chemotherapy-naïve mCRPC (COU-AA-302) [26] and post-docetaxel mCRPC (COU-AA-301) [27]. Its role was further expanded by the LATITUDE and STAMPEDE trials, which established improved survival outcomes in high-risk metastatic castration-sensitive prostate cancer (mCSPC), thereby shifting treatment paradigms toward earlier and more intensive AR-axis suppression [28,29,30].

**Figure 1:**
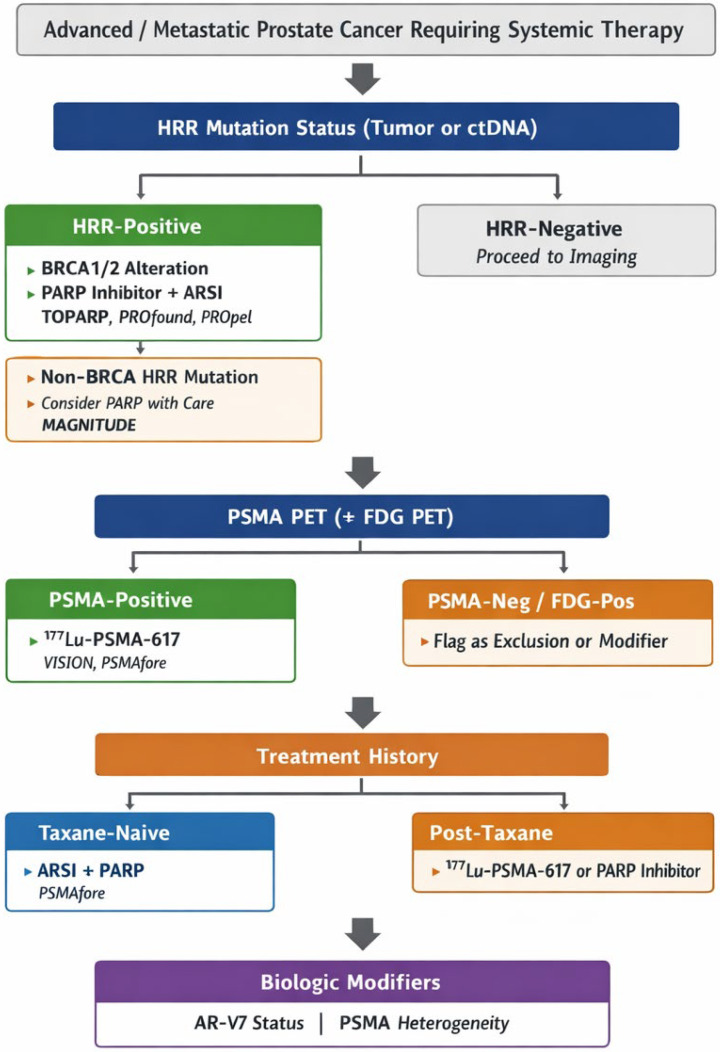
Prostate cancer clinical decision algorithm, where; ARSI—androgen receptor signaling inhibitor; AR-V7—androgen receptor splice variant 7; BRCA1/2—breast cancer susceptibility gene 1 or 2; ctDNA—circulating tumor DNA; FDG PET—^18^F-fluorodeoxyglucose positron emission tomography; HRR—homologous recombination repair; PARP—poly(ADP-ribose) polymerase; PET—positron emission tomography; PSMA—prostate-specific membrane antigen; ^177^Lu-PSMA-617—lutetium-177–labeled PSMA-617 radioligand.

Despite these advances, critical clinical questions remain unresolved. Optimal sequencing of PARP inhibitors, PSMA-directed RLT, and next-generation AR pathway inhibitors has not been fully defined, particularly as these agents move into earlier lines of therapy. Combination strategies—including PARP inhibitors plus ARSIs or the integration of RLT with systemic treatments—are actively being explored, yet their appropriate application remains the subject of ongoing investigation and debate [31,32,33,34]. In parallel, real-world implementation is influenced by access to biomarker testing, PSMA PET imaging, and multidisciplinary expertise, raising important considerations regarding equity and generalizability of care [35,36]. Figure 1 illustrates the clinical decision-making algorithm for prostate cancer.

In this context, the present review aims to extend beyond existing narrative summaries by providing an integrative, biomarker-driven synthesis across these three therapeutic pillars, with a particular emphasis on therapeutic sequencing rather than isolated drug classes. We highlight how genomic profiling, functional imaging, prior treatment exposure, and toxicity considerations intersect to inform clinical decision-making. In addition, we discuss future directions and unresolved questions, including patient selection, combination strategies, and long-term safety. By framing these therapies within a unified clinical context, this review seeks to clarify their complementary roles and to support rational, personalized management of advanced prostate cancer.

This synthesis is based on a narrative literature review of PubMed and Scopus using the search terms “olaparib,” “lutetium (^177^Lu) vipivotide tetraxetan,” and “abiraterone” in combination with “targeted therapy” and “prostate cancer.” Peer-reviewed articles published between 2005 and 2025 were selected according to relevance, methodological rigor, and contribution to understanding therapeutic efficacy, mechanisms of action, resistance, and clinical integration. Both preclinical and clinical studies were included when mechanistic insights informed therapeutic strategy, enabling a comprehensive assessment of the benefits and limitations of targeted therapies in contemporary prostate oncology.

## 2. Olaparib

**Figure 2:**
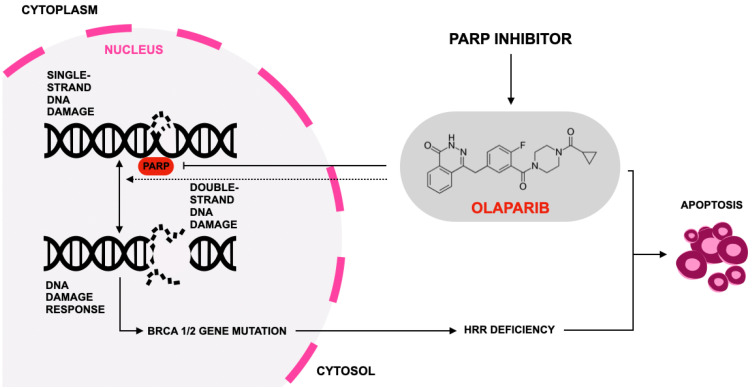
Mechanism of action of olaparib according to [41]. Olaparib, a poly(ADP-ribose) polymerase (PARP) inhibitor, blocks PARP enzymes involved in the repair of single-strand DNA breaks. Inhibition of PARP leads to accumulation of unrepaired single-strand lesions, which are converted into double-strand DNA breaks during DNA replication. Tumor cells harboring BRCA1/2 mutations or HRD are unable to effectively repair these double-strand breaks through the HRR pathway. The resulting accumulation of irreparable DNA damage induces cell death, thereby selectively targeting cancer cells with defects in DNA repair mechanisms. All abbreviations employed are defined in the text in the Abbreviations Section.

Olaparib is an orally available small-molecule inhibitor of PARP enzymes that has transformed treatment paradigms for tumors with defective HRR, most notably cancers harboring BRCA1/2 alterations; by binding the catalytic domain of PARP1 and PARP2, olaparib prevents PARylation at sites of single-strand DNA breaks, promotes trapping of PARP on DNA, converts replication-associated single-strand breaks into lethal double-strand breaks, and thereby induces synthetic lethality in cells deficient in HRR machinery (Figure 2) [37,38,39,40]. In clinical practice, this mechanistic specificity underpins the rationale for biomarker-guided use of olaparib and highlights the importance of accurate identification of HRR defects prior to treatment initiation.

The biochemical selectivity for PARP1/2 and the dual capacity of olaparib to inhibit catalytic activity and stabilize PARP–DNA complexes underlie both its antitumor efficacy and its class-typical toxicities, including myelosuppression and gastrointestinal adverse effects [42,43]. Early clinical development focused on BRCA-mutant ovarian and breast cancers, culminating in the first regulatory approvals in BRCA-mutated ovarian cancer in 2014 and establishing proof of principle for therapeutic exploitation of HRR deficiency [44,45]. Subsequent mechanistic and translational research broadened the understanding of PARP inhibition by elucidating additional modes of action—such as effects on replication-fork stability and immunomodulatory consequences—and by clarifying why specific HRR gene alterations (e.g., BRCA1/2) predict greater sensitivity than others (e.g., ATM), while also defining biologically plausible mechanisms of acquired resistance, including reversion mutations, restoration of end resection, replication-fork protection, and drug efflux [46,47,48]. These insights have direct implications for real-world treatment durability and for the strategic sequencing of therapies to delay or circumvent resistance.

Interest in olaparib for prostate cancer arose from the recognition that approximately 20–30% of mCRPC harbor deleterious germline or somatic alterations in HRR genes, defining a clinically actionable molecular subset [49,50]. Early clinical development of PARP inhibitors in this setting was characterized by exploratory, biomarker-unselected phase II studies, most notably TOPARP-A, which demonstrated that antitumor activity was strongly enriched among patients with DNA-repair defects. In TOPARP-A, objective responses and durable clinical benefit were concentrated in tumors harboring deleterious BRCA2, ATM, CHEK2, and PALB2 alterations, thereby validating a biomarker-led treatment strategy and establishing proof of concept for PARP inhibition in prostate cancer [42,51]. Parallel analyses confirmed meaningful improvements in rPFS and OS among HRR-altered tumors, directly informing the design of subsequent molecularly enriched trials [12,13].

Prospective validation followed in TOPARP-B, which restricted enrollment to patients with predefined HRR mutations and employed a randomized dose-comparison design, demonstrating higher response rates and greater clinical benefit in BRCA1/2-altered tumors compared with other HRR subgroups [14]. These findings were definitively confirmed in the phase III PROfound trial, which enrolled men with HRR-mutated mCRPC who had progressed on enzalutamide or abiraterone and used rPFS assessed by blinded independent central review as the primary endpoint. In PROfound, olaparib significantly improved rPFS compared with physician’s choice of androgen receptor–targeted therapy, increasing median rPFS from 3.0 to 7.4 months (absolute gain 4.4 months; HR 0.34; 95% CI: 0.25–0.47), and also prolonged OS (19.1 vs. 14.7 months; HR 0.69; 95% CI: 0.50–0.97), despite substantial crossover [12]. Benefit was particularly pronounced in patients with BRCA1/2 and ATM alterations, establishing PARP inhibition as a standard of care in this molecularly defined population and supporting regulatory approval in prostate cancer [42,44,52].

Regulatory milestones followed rapidly. On 19 May 2020, the U.S. Food and Drug Administration approved olaparib (LYNPARZA^®^) for adult patients with deleterious or suspected deleterious germline or somatic HRR gene–mutated mCRPC following progression on enzalutamide or abiraterone, with companion diagnostic assays specified to identify eligible patients; subsequent regulatory refinements expanded indications and enabled combination strategies in selected genomic subgroups [53,54,55]. These developments reinforced the practical necessity of integrating timely germline and somatic HRR testing into standard clinical workflows.

As clinical development progressed, attention increasingly shifted toward combination strategies and broader patient populations. Preclinical evidence of reciprocal interactions between AR signaling and DNA repair pathways provided a rationale for combining PARP inhibitors with androgen receptor signaling inhibitors (ARSIs). In this context, first-line randomized trials such as PROpel and MAGNITUDE evaluated PARP inhibitor–abiraterone combinations. In PROpel, olaparib plus abiraterone significantly prolonged rPFS compared with abiraterone alone (24.8 vs. 16.6 months; absolute gain 8.2 months; HR 0.66; 95% CI: 0.54–0.81), with final OS analysis showing a numerical improvement (42.1 vs. 34.7 months; HR 0.81; 95% CI: 0.67–1.00). Exploratory analyses suggested activity beyond BRCA1/2-mutated tumors; however, the magnitude of benefit was greatest in patients with HRR alterations, particularly BRCA mutations [26,56]. These findings supported FDA approval of the combination for BRCA-mutated mCRPC in 2023 [57,58,59].

In contrast, the MAGNITUDE trial prospectively stratified patients by HRR status and failed to demonstrate benefit in HRR-negative disease, leading to early closure of this cohort. This outcome underscored important biological and clinical distinctions between HRR-defined subpopulations and cautioned against extrapolating benefit to biomarker-unselected patients [18,19,20]. Collectively, these data indicate that while PARP inhibitor–ARSI combinations represent a significant therapeutic advance for patients with defined HRR alterations—particularly BRCA1/2 mutations—their routine use outside molecularly selected populations remains controversial [27,28].

Across clinical trials, efficacy has consistently correlated with specific HRR genotypes. Patients with BRCA1/2 alterations derive the most pronounced and durable improvements in rPFS and OS, whereas alterations in ATM, CHEK2, or CDK12 confer more variable and generally modest benefit. These genotype–phenotype associations have informed guideline recommendations advocating routine germline and somatic HRR testing in metastatic disease to guide PARP inhibitor use and familial risk counseling [47,60,61]. Implementation guidance has further addressed assay selection, interpretation of variants of uncertain significance, and concordance between tissue- and plasma-based testing modalities, all of which remain critical for real-world application [62].

The safety profile of olaparib is well characterized. Across pivotal trials including TOPARP-A, TOPARP-B, PROfound, and PROpel, grade ≥3 adverse events were reported in approximately 40–55% of patients, depending on line of therapy and combination partner [63,64,65,66]. Hematologic toxicity, particularly anemia, represented the most prominent severe adverse event, with grade ≥3 anemia occurring in approximately 20–25% of patients receiving olaparib monotherapy and 15–20% of those treated with olaparib plus abiraterone [67,68,69]. Fatigue and asthenia were common, although grade ≥3 events were generally infrequent (<10%), and gastrointestinal toxicities such as nausea and vomiting were typically manageable. These adverse events were often cumulative and frequently necessitated dose interruptions or reductions, underscoring the importance of routine hematologic monitoring and early supportive management, particularly in heavily pretreated or frail populations [67,68,69,70]. In combination regimens, increased rates of lymphopenia and venous thromboembolism have been reported, while rare but serious risks of myelodysplastic syndrome and acute myeloid leukemia necessitate long-term hematologic surveillance [67,68,69].

Despite robust efficacy in selected populations, acquired resistance remains a major limitation to durable disease control. Identified mechanisms include BRCA1/2 reversion mutations, replication-fork stabilization, altered PARP expression, drug efflux, and adaptive DNA-damage signaling networks, driving ongoing efforts to optimize sequencing and develop rational combination strategies to minimize cross-resistance [48,60]. Accordingly, current guidelines integrate PARP inhibitors into biomarker-driven treatment algorithms while emphasizing practical challenges related to genomic testing access, toxicity management, and sequencing relative to other life-prolonging therapies [61,71].

**Figure 3:**
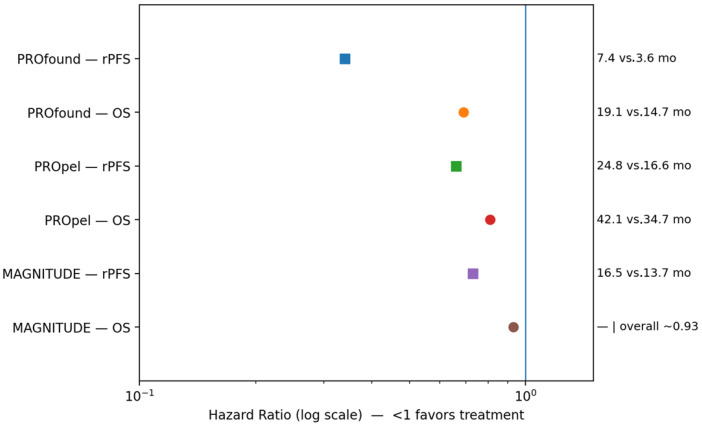
Comparative rPFS and OS outcomes for olaparib across prostate cancer disease states; forest–bar plots display hazard ratios and absolute median outcomes for rPFS and OS from pivotal phase II–III trials; panel shows outcomes for olaparib-based therapy in homologous recombination repair–selected and all-comer mCRPC, and the blue vertical line is the reference line at HR = 1.0 (no difference between treatment and control), where HR—hazard ratio; OS—overall survival; rPFS—radiographic progression-free survival.

The clinical development of olaparib exemplifies precision oncology in prostate cancer: a therapy whose chemically defined mechanism targets a specific molecular vulnerability, whose advancement progressed from exploratory biomarker-unselected studies to definitive randomized trials, and whose continued evolution—through refined diagnostics, combination strategies, and resistance-focused research—continues to shape real-world treatment paradigms for advanced disease [51,52,53,54]. Table 1 summarizes treatment-emergent adverse events associated with olaparib and recommended management strategies; Table 2 presents key pivotal clinical trials evaluating its efficacy and safety; and Figure 3 illustrates comparative rFPS and OS outcomes for olaparib across prostate cancer disease states.

## 3. Lutetium (^177^Lu) Vipivotide Tetraxetan

Lutetium (^177^Lu) vipivotide tetraxetan (also known as ^177^Lu-PSMA-617, marketed as Pluvicto) is a radioligand therapeutic agent that delivers targeted β-radiation to cells expressing the transmembrane protein PSMA, which is highly overexpressed on the surface of most prostate cancer cells but has limited expression in normal tissues, thereby enabling selective tumor targeting with relative sparing of nonmalignant organs [82,83,84]. This biologic specificity has made ^177^Lu-PSMA-617 a cornerstone of PSMA-directed “theranostic” strategies and underscores the central importance of accurate biomarker-based patient selection in routine clinical practice.

**Figure 4:**
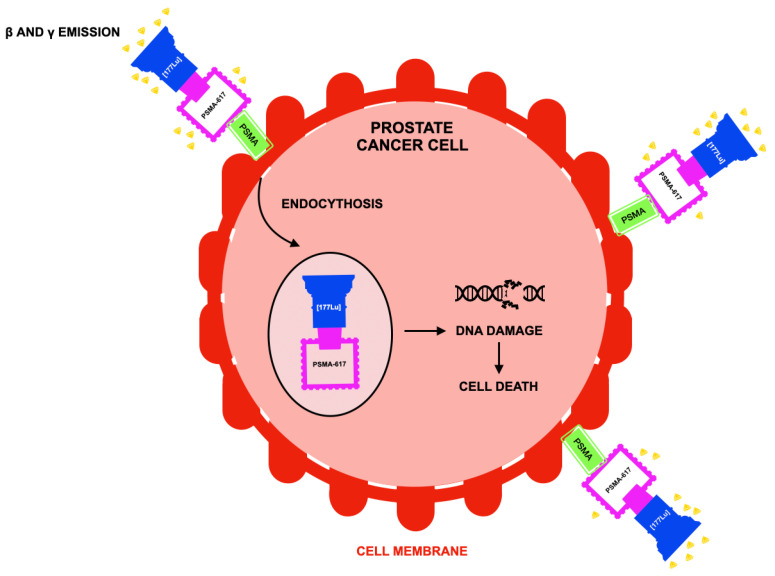
Mechanism of action of Lutetium (^177^Lu) vipivotide tetraxetan according to [92]. Lutetium-177 vipivotide tetraxetan is a radioligand therapy composed of the PSMA-617 targeting ligand labeled with the radioactive isotope lutetium-177 (^177^Lu). After systemic administration, PSMA-617 selectively binds to prostate-specific membrane antigen (PSMA) expressed on the surface of prostate cancer cells and is subsequently internalized. The ^177^Lu radionuclide emits β-particles, which cause localized DNA damage, including double-strand breaks, in targeted tumor cells and neighboring cells, as well as γ-radiation that enables imaging and dosimetry. The resulting accumulation of radiation-induced DNA damage leads to tumor cell death while limiting exposure to the surrounding normal tissues. All abbreviations employed are defined in the text in the Abbreviations section.

Following intravenous administration, the PSMA-binding ligand vipivotide tetraxetan binds with high affinity to PSMA on prostate cancer cells, undergoes internalization, and delivers the conjugated radioisotope Lutetium-177, whose emitted β-particles induce DNA damage—particularly double-strand breaks—leading to tumor cell death. The relatively short tissue path length of β-particles enables a beneficial “cross-fire” effect, whereby adjacent tumor cells with lower or heterogeneous PSMA expression may also receive cytotoxic radiation, partially mitigating intratumoral heterogeneity [85,86,87,88]. This mechanism distinguishes RLT from both conventional systemic treatments and external-beam radiotherapy, allowing repeated systemic administration with molecularly targeted cytotoxicity (Figure 4) [89,90,91]. However, heterogeneity of PSMA expression across and within metastatic lesions remains a clinically relevant limitation, influencing both response durability and resistance.

The clinical development and regulatory approval of lutetium (^177^Lu) vipivotide tetraxetan (Pluvicto^®^) represent a major advance in PSMA-directed radioligand therapy (RLT) for mCRPC. The evidence base supporting PSMA-targeted RLT has been built on progressively refined patient selection, imaging-based eligibility criteria, and robust efficacy endpoints. Early phase II and single-arm studies established proof of concept by enrolling PSMA-positive mCRPC patients and demonstrating meaningful PSA responses with manageable toxicity, thereby providing the biological and clinical rationale for further development and directly informing the design of subsequent randomized trials incorporating stringent molecular imaging selection [21,22].

The first major regulatory milestone for PSMA–targeted radioligand therapy occurred on 23 March 2022, when the U.S. Food and Drug Administration (FDA) approved ^177^Lu-PSMA-617 for adult patients with PSMA-positive mCRPC who had progressed following androgen receptor pathway inhibitor (ARPI) therapy and taxane-based chemotherapy. This approval was based on the phase III VISION trial, which mandated PSMA positivity on PET imaging and excluded patients with discordant PSMA-negative/FDG-positive disease, thereby ensuring molecularly appropriate patient selection [93,94,95,96]. In VISION, treatment with ^177^Lu-PSMA-617 plus best standard of care significantly improved both rPFS and OS compared with standard of care alone. Median rPFS increased from 3.4 to 8.7 months (HR 0.40; 95% CI: 0.29–0.56), while median OS improved from 11.3 to 15.3 months (HR 0.62; 95% CI: 0.52–0.74), with consistent benefit across secondary endpoints including objective response and time to symptomatic skeletal events [21,85,87,97]. Importantly, these survival gains were achieved without deterioration in quality-of-life metrics, despite higher rates of grade ≥3 adverse events in the treatment arm [85,98,99].

Additional trials further refined patient selection and comparative efficacy. The randomized phase II TheraP trial mandated dual-tracer PSMA and FDG PET imaging and compared ^177^Lu-PSMA-617 with cabazitaxel, using PSA response as the primary endpoint. TheraP demonstrated superior biochemical response rates and a more favorable toxicity profile for radioligand therapy, reinforcing the critical role of imaging-based enrichment strategies and establishing PSMA PET as an essential biomarker for treatment eligibility and outcome optimization [22]. The therapeutic positioning of ^177^Lu-PSMA-617 has continued to evolve toward earlier disease settings. In the phase III PSMAfore trial, conducted in taxane-naïve patients with PSMA-positive mCRPC who had progressed on a single prior ARPI, radioligand therapy significantly prolonged rPFS compared with switching to an alternative AR-targeted agent, with an acceptable safety profile [100,101,102,103]. These findings led to FDA expansion of the U.S. indication on 28 March 2025 to include patients appropriate for delaying taxane-based chemotherapy [104]. In contrast, regulatory approvals remain region specific: the European Medicines Agency granted marketing authorization on 9 December 2022 for post-taxane mCRPC, while pre-taxane use remains outside the current European label [105]. Real-world evidence has reinforced the external validity of these findings, with retrospective analyses of heavily pretreated mCRPC populations treated outside clinical trials demonstrating PSA response rates comparable to those observed in VISION, supporting the feasibility and effectiveness of PSMA-directed therapy in routine oncology practice [105,106].

Pharmacokinetic, biodistribution, and dosimetry studies—including dedicated substudies within VISION—have provided critical insights for clinical implementation. Following intravenous administration, ^177^Lu-PSMA-617 rapidly distributes to PSMA-expressing tumors and to physiologic uptake sites including kidneys, liver, salivary and lacrimal glands, bladder wall, bone marrow, gastrointestinal tract, and other organs, and is predominantly cleared via the renal route with bi-exponential clearance kinetics. Reported effective half-lives include an initial component of approximately 1.7 ± 0.8 h and a terminal half-life of 41.1 ± 9.3 h [107,108,109]. Organs receiving the highest absorbed radiation doses include the salivary glands and kidneys, as well as the bladder wall, large intestine, lacrimal glands, and rectum, thereby defining dose-limiting organs and informing radiation safety planning and cycle limits [107,108,109]. These dosimetry findings also support the use of PSMA PET–based intensity thresholds for eligibility, as applied in VISION and PSMAfore, where sufficient tumor uptake relative to reference organs was required to ensure favorable tumor-to-organ dose ratios. Because renal handling is central to clearance, renal function and cumulative radiation exposure remain key considerations in patient selection and treatment planning [108,110].

Across clinical trials and post-marketing experience, the safety profile of ^177^Lu-PSMA-617 is characterized by a toxicity spectrum distinct from that of systemic hormonal or targeted therapies. The most common adverse reactions (≥20% of treated patients) include fatigue, xerostomia, nausea, anemia, decreased appetite, and constipation, while the most frequent laboratory abnormalities (≥30%) include lymphopenia, reductions in hemoglobin, leukocytes, and platelets, as well as decreases in serum calcium and sodium [82,85]. In VISION and PSMAfore, grade ≥3 adverse events were reported in approximately 50% of treated patients [100,101,102], and in VISION specifically, grade 3–4 adverse events occurred in approximately 52.7% of patients receiving ^177^Lu-PSMA-617 versus 38.0% in the control arm, although patient-reported quality-of-life measures were largely preserved [85]. Xerostomia and salivary gland toxicity are characteristic adverse effects, occurring in approximately 30–60% of patients but predominantly grade 1–2 and infrequently dose-limiting [21,82,85,111]. In contrast, bone marrow suppression constitutes the principal severe toxicity, with grade ≥3 anemia observed in approximately 10–13% of patients, alongside thrombocytopenia and neutropenia, particularly in individuals with extensive osseous metastatic burden [21,85,105,111]. These observations emphasize careful patient selection, baseline marrow reserve assessment, and long-term hematologic surveillance.

Because ^177^Lu-PSMA-617 is a radiopharmaceutical, cumulative radiation exposure introduces long-term considerations, including renal toxicity, sustained marrow suppression, embryo–fetal toxicity, potential fertility impairment, and theoretical genotoxic, carcinogenic, or secondary malignancy risks, all of which require counseling and longitudinal monitoring [112,113]. Dosimetry analyses further indicate that patients with impaired renal function may receive up to a two-fold higher absorbed kidney dose compared with those with normal creatinine clearance, increasing the likelihood of approaching renal safety thresholds after multiple cycles; accordingly, baseline renal function and bone marrow reserve cut-offs are emphasized as critical eligibility criteria to mitigate nephrotoxicity and cumulative marrow suppression [108,110]. In addition, discordant FDG-positive/PSMA-negative lesions—excluded in VISION and linked to inferior outcomes—have emerged as a clinically meaningful modifier of treatment suitability, reflecting biologically aggressive PSMA-non-avid disease unlikely to benefit from PSMA-targeted radioligand therapy and reinforcing the value of dual-tracer imaging for contemporary patient selection [93,94,95,96].

Despite its targeted mechanism, resistance to ^177^Lu-PSMA-617 can emerge through loss or downregulation of PSMA expression, selection of PSMA-negative tumor clones, limited radiation delivery to bulky or poorly perfused lesions, or intact DNA-damage repair capacity, underscoring the need for rational sequencing and combination strategies to maximize durable benefit. Clinically, lutetium (^177^Lu) vipivotide tetraxetan represents a paradigm shift in prostate cancer management by integrating molecular imaging with targeted systemic radiotherapy, embodying a theranostic approach that uses PSMA PET for patient selection and targeted radiotherapy for disease control [86,89,113,114]. Its expanding role reflects not only demonstrated survival benefit but also its potential to delay chemotherapy or serve as a therapeutic bridge between hormonal therapy and cytotoxic regimens in appropriately selected patients [115,116,117,118]. Ongoing trials are exploring earlier disease settings, optimized dosing and fractionation schedules, and combination approaches with AR blockade, PARP inhibitors, immunotherapy, and chemotherapy [119,120,121,122]. Emerging predictive dosimetry strategies—including physiologically based pharmacokinetic modeling and machine-learning–assisted dose optimization—may further refine patient selection and improve the therapeutic index, particularly in patients with heterogeneous tumor burden or compromised organ function [119,123,124].

**Figure 5:**
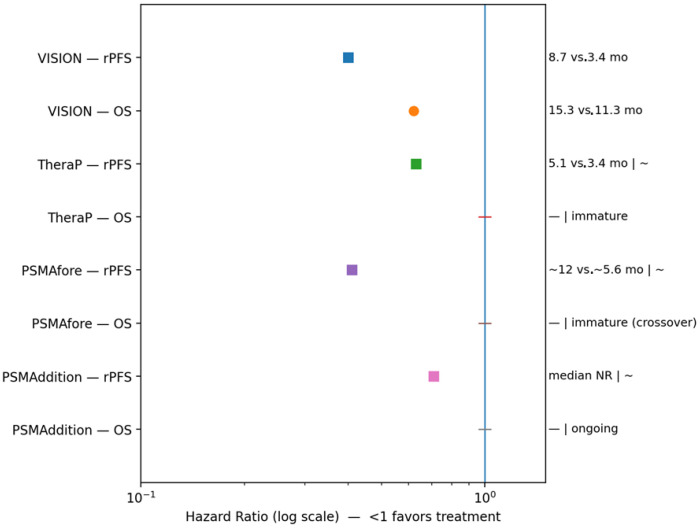
Comparative rPFS and OS outcomes for ^177^Lu-PSMA-617 across prostate cancer disease states; forest–bar plots display hazard ratios and absolute median outcomes for radiographic rPFS and OS from pivotal phase II–III trials; panel summarizes efficacy of ^177^Lu-PSMA-617 across PSMA-positive disease states, including heavily pretreated mCRPC and earlier-line settings, and the blue vertical line is the reference line at HR = 1.0 (no difference between treatment and control), where HR—hazard ratio; OS—overall survival; rPFS—radiographic progression-free survival; NR—not reached.

^177^Lu-PSMA-617 is therefore a PSMA-targeted radioligand therapy whose development has progressed from early proof-of-concept studies to definitive randomized trials and region-specific regulatory approvals, establishing it as a central component of precision oncology for mCRPC. Continued optimization will depend on refined imaging-based selection, individualized dosimetry, strategic sequencing with other life-prolonging therapies, and deeper understanding of resistance mechanisms. Table 3 summarizes treatment-emergent adverse events and management strategies; Table 4 highlights pivotal clinical trials evaluating efficacy and safety; and Figure 5 shows comparative rPFS and OS outcomes for ^177^Lu-PSMA-617 across prostate cancer disease states.

## 4. Abiraterone

**Figure 6:**
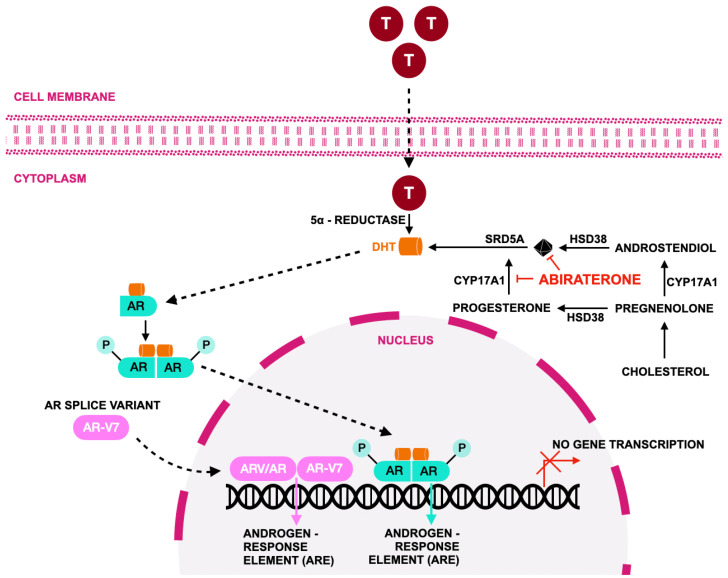
Mechanism of action of abiraterone according to [41]. Abiraterone acetate is an androgen biosynthesis inhibitor that suppresses androgen signaling by targeting cytochrome P450 17A1 (CYP17), an enzyme with both 17α-hydroxylase and C17,20-lyase activity that is essential for androgen production. Inhibition of CYP17 reduces the synthesis of testosterone and dihydrotestosterone (DHT) in the testes, adrenal glands, and tumor tissue. The resulting depletion of circulating and intratumoral androgens limits activation of the AR, including full-length AR and constitutively active splice variants such as AR-V7, thereby impairing downstream AR signaling and prostate cancer cell growth. All abbreviations employed are defined in the text in the Abbreviations section.

Abiraterone acetate is an orally administered, selective, irreversible inhibitor of cytochrome P450 17α-hydroxylase/C17,20-lyase (CYP17A1), a critical enzyme in androgen biosynthesis, and is therefore classified as an androgen-biosynthesis–blocking androgen-receptor pathway inhibitor that suppresses extragonadal, intratumoral, and testicular androgen production beyond that achieved with conventional gonadal suppression alone [134,135,136]. By targeting CYP17A1 at a key steroidogenic branch point, abiraterone effectively inhibits the synthesis of dehydroepiandrosterone and androstenedione, the principal precursors of testosterone and dihydrotestosterone (DHT). This mechanism enables sustained androgen deprivation even in castrate conditions, where residual androgen production continues to drive tumor progression (Figure 6) [137,138,139]. In real-world clinical practice, this profound androgen suppression underpins abiraterone’s broad applicability across multiple disease states but also shapes considerations regarding sequencing with other androgen-receptor–directed therapies.

Pharmacologically, abiraterone blocks the conversion of pregnenolone and progesterone into their 17α-hydroxylated derivatives and downstream androgen precursors, resulting in marked reductions in serum testosterone levels in men with castration-resistant prostate cancer (CRPC) [140,141,142]. Preclinical enzymology studies first demonstrated near-complete abrogation of androgen synthesis with selective CYP17A1 inhibition, and early-phase clinical investigations by Attard and colleagues subsequently confirmed that this biochemical suppression translated into clinically meaningful PSA declines in heavily pretreated CRPC patients [143,144,145]. These foundational data established both the biological rationale and clinical feasibility of sustained androgen biosynthesis inhibition.

The clinical evidence supporting abiraterone across the prostate cancer disease continuum is derived from rigorously designed trials with clearly defined patient populations and survival-focused endpoints. Early phase II single-arm studies in mCRPC enrolled heterogeneous cohorts with variable prior therapy exposure and demonstrated consistent PSA and radiographic responses, establishing proof of activity and tolerability in both pre- and post-chemotherapy settings and directly informing the design of subsequent phase III trials with more stringent eligibility criteria and clinically meaningful primary endpoints [26,27].

The first pivotal randomized evidence supporting abiraterone’s survival benefit emerged from the COU-AA-301 trial, a global phase III study conducted in men with post-docetaxel mCRPC. In this setting, abiraterone acetate plus prednisone significantly improved OS, with a median OS of 15.8 months compared with 11.2 months for placebo (absolute gain 4.6 months; HR 0.74; 95% CI: 0.64–0.86), and also prolonged rPFS (median 5.6 vs. 3.6 months; HR 0.66; 95% CI: 0.58–0.76). Additional benefits included higher PSA response rates, improved pain control, and delayed radiographic progression, leading to regulatory approval and establishing abiraterone as a standard post-chemotherapy option [146,147,148].

This benefit was subsequently extended to chemotherapy-naïve mCRPC in the COU-AA-302 trial, a randomized, double-blind phase III study with co-primary endpoints of OS and rPFS. Abiraterone plus prednisone prolonged median OS to 34.7 months compared with 30.3 months for placebo (absolute gain 4.4 months; HR 0.81; 95% CI: 0.70–0.93) and significantly delayed radiographic progression (HR 0.43; 95% CI: 0.35–0.52), while also extending time to opiate use and preserving patient-reported quality of life [149,150,151]. Together, these findings confirmed that androgen biosynthesis remains a clinically relevant therapeutic target even after the development of castration resistance.

Patient selection was further broadened to earlier disease states in the LATITUDE and STAMPEDE trials. LATITUDE demonstrated that adding abiraterone to androgen deprivation therapy (ADT) in patients with high-risk metastatic hormone-sensitive prostate cancer significantly improved rPFS (30.7 vs. 18.3 months; HR 0.53; 95% CI: 0.37–0.76) and OS (HR 0.62; 95% CI: 0.51–0.76), yielding substantial absolute survival gains [152,153,154,155]. Similarly, the multi-arm STAMPEDE platform trial showed that intensification of ADT with abiraterone significantly improved OS compared with ADT alone, with hazard ratios ranging from approximately 0.62 to 0.72 and absolute median OS gains of roughly 12–16 months in metastatic subgroups [29]. Subsequent analyses confirmed consistent benefit across both low- and high-volume metastatic disease, reinforcing abiraterone’s versatility and informing real-world treatment algorithms across diverse patient populations [156,157,158,159].

Real-world evidence has corroborated these trial results, demonstrating comparable outcomes in broader and more heterogeneous populations, including older patients and those with cardiovascular comorbidities or extensive prior therapy [160,161,162]. Translational studies have identified biomarkers that may refine patient selection and sequencing decisions, most notably the AR splice variant AR-V7 detected in circulating tumor cells, which has been associated with reduced responsiveness to abiraterone. These observations support biomarker-driven stratification to guide transitions to alternative treatment classes such as taxanes or PSMA-targeted radioligand therapy, although AR-V7 testing is not yet routinely implemented [160,161,162].

Abiraterone’s safety profile reflects its mechanism of CYP17-mediated adrenal steroidogenesis inhibition. Suppression of cortisol synthesis results in compensatory adrenocorticotropic hormone elevation and mineralocorticoid excess, manifesting clinically as hypertension, hypokalemia, and fluid retention, with occasional cardiac arrhythmias. Across pivotal trials including COU-AA-301, COU-AA-302, and LATITUDE, grade ≥3 adverse events were reported in approximately 40–55% of patients [26,27,28]. Grade ≥3 hypertension occurred in approximately 10–20% of patients, while grade ≥3 hypokalemia was observed in 5–10%; fluid retention and edema were common but predominantly low grade [163,164,165]. These toxicities typically occurred early during treatment and were generally manageable with routine coadministration of corticosteroids, antihypertensive therapy, electrolyte monitoring, and dose modification when required. Hepatic enzyme elevations, fatigue, and infrequent but clinically significant hepatotoxicity necessitate regular laboratory surveillance [166,167]. Cardiovascular toxicity remains a key consideration, particularly in patients with preexisting cardiovascular disease, and often influences treatment selection and sequencing when alternative androgen receptor–directed or non-hormonal therapies are available [168,169].

Despite its established efficacy, resistance to abiraterone inevitably develops. Mechanisms include AR amplification or mutation, intratumoral androgen synthesis bypassing CYP17 inhibition, activation of alternative steroidogenic pathways, and emergence of constitutively active AR splice variants such as AR-V7. These resistance mechanisms limit response durability and contribute to cross-resistance with other androgen receptor–directed therapies, underscoring the importance of timely transition to mechanistically distinct treatments such as chemotherapy, PARP inhibitors, or PSMA-targeted radioligand therapy [170,171,172,173].

Combination strategies have therefore been extensively explored. Abiraterone has been evaluated in combination with PARP inhibitors, next-generation AR antagonists, radiopharmaceuticals, and immunotherapeutic agents, with preclinical and early clinical data suggesting potential synergy through enhanced DNA-damage susceptibility and modulation of the tumor microenvironment [170,171,172,173,174,175]. However, negative results from trials such as CYCLONE 2, which failed to demonstrate benefit from adding the CDK4/6 inhibitor abemaciclib to abiraterone in an unselected mCRPC population, highlight the necessity of strong biological rationale and appropriate patient selection when extending combination approaches [31].

Collectively, these studies illustrate how differences in trial design, patient population, biomarker integration, and endpoint selection have shaped the optimal clinical positioning and sequencing of abiraterone-based therapy. Abiraterone acetate exemplifies mechanism-driven treatment in advanced prostate cancer, with extensive validation across hormone-sensitive and castration-resistant settings and continued relevance as a therapeutic backbone within evolving multimodal strategies. As treatment landscapes increasingly incorporate PARP inhibitors, PSMA-targeted radioligand therapy, and next-generation androgen receptor blockade, abiraterone’s role will continue to be refined through biomarker-driven patient selection, rational sequencing, and resistance-aware treatment strategies [176,177,178,179].

**Figure 7:**
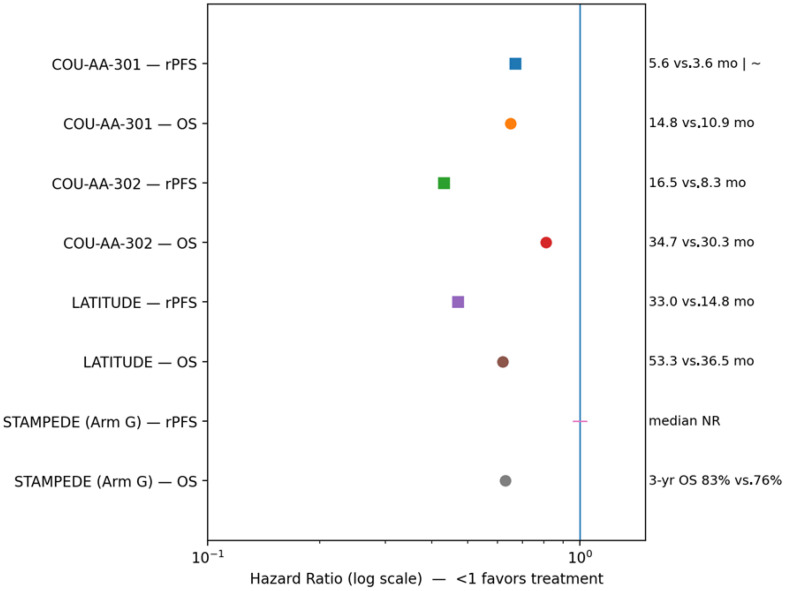
Comparative rPFS and OS outcomes for abiraterone across prostate cancer disease states; forest–bar plots display hazard ratios and absolute median outcomes for rPFS and OS from pivotal phase II–III trials; panel illustrates outcomes with abiraterone plus prednisone across the disease continuum, demonstrating the largest absolute OS benefit when used in hormone-sensitive disease, and the blue vertical line is the reference line at HR = 1.0 (no difference between treatment and control), where HR—hazard ratio; OS—overall survival; rPFS—radiographic progression-free survival; NR—not reached.

Intensification of AR signaling inhibition with abiraterone confers substantial survival benefit across metastatic hormone-sensitive and castration-resistant prostate cancer, with hazard ratios ranging from approximately 0.62 to 0.81 and absolute median OS gains of roughly 4–22 months depending on disease setting and baseline risk [26,27,28]. Abiraterone remains a cornerstone of contemporary precision oncology for prostate cancer, supported by a well-characterized mechanism, robust survival benefit, and a manageable safety profile. Ongoing research continues to optimize sequencing, overcome resistance, and extend benefit to a broader range of patients. Table 5 summarizes treatment-emergent adverse events and recommended management strategies; Table 6 highlights key pivotal clinical trials assessing efficacy and safety; and Figure 7 presents comparative rPFS and OS outcomes for abiraterone across prostate cancer disease states.

## 5. Anticipated Developments

Future therapeutic strategies in prostate cancer increasingly emphasize precision oncology, convergence of biological pathways, and multimodal integration, reflecting a fundamental shift toward treatments that are mechanistically informed yet dynamically adaptable to tumor evolution. Advances in PARP inhibition, PSMA-targeted RLT, and next-generation androgen-receptor (AR) blockade now constitute the conceptual and clinical backbone of emerging therapeutic frameworks, each grounded in a deeper understanding of resistance biology and disease heterogeneity [184,185]. Despite these advances, their real-world implementation remains constrained by important limitations related to toxicity burden, access to specialized diagnostics and infrastructure, financial cost, and persistent gaps in clinical evidence, underscoring the need for continued critical evaluation alongside innovation.

Expanding translational research efforts are increasingly focused on linking molecular phenotypes with therapeutic vulnerabilities to enable rational combinations across treatment classes. For example, ongoing work suggests that combined PARP inhibition and androgen-signaling suppression may ultimately extend beyond canonical BRCA1/2- or HRR-mutant populations. This evolution is driven by increasingly sophisticated genomic, epigenomic, and functional biomarker analyses that define synthetic-lethal interactions with greater granularity [186,187]. However, widespread clinical adoption of such approaches is currently limited by the lack of standardized assays, variable reproducibility across laboratories, and uncertainty regarding how best to integrate emerging biomarkers into routine treatment algorithms.

Novel biomarker platforms—including genomic scarring signatures that reflect cumulative homologous recombination deficiency, RAD51 foci assays that provide functional readouts of HRR activity, and circulating tumor DNA profiling that captures intrapatient heterogeneity over time—offer promising tools for more precise patient stratification [188,189,190,191,192,193,194]. While these technologies hold significant potential to expand the therapeutic reach of agents such as olaparib, they also raise unresolved questions regarding cost-effectiveness, regulatory validation, and optimal thresholds for clinical decision-making. Prospective trials incorporating these biomarkers as stratification or enrichment tools will be essential to determine their true clinical utility.

Radioligand therapy, including Lutetium (^177^Lu) vipivotide tetraxetan, represents one of the most transformative advances in metastatic prostate cancer management, with compelling evidence of PSA responses, radiographic improvement, and survival benefit even in heavily pretreated disease. Nonetheless, its broader adoption is limited by logistical complexity, dependence on PSMA PET imaging, radiation-safety infrastructure requirements, and variable global availability. Current trials exploring earlier use of RLT and combinations with hormonal intensification or DNA repair–modulating agents are biologically compelling, particularly given largely non-overlapping toxicity profiles and potential synergy through immune activation and amplified DNA damage [195,196,197,198,199]. However, unanswered questions remain regarding cumulative radiation exposure, optimal dose fractionation, long-term renal and marrow toxicity, and cost sustainability—issues that must be addressed before widespread front-line implementation can be justified.

Next-generation AR pathway inhibitors and rational combination strategies remain central to both current practice and future innovation, driven by detailed characterization of resistance mechanisms such as AR amplification, constitutively active splice variants, epigenetically driven lineage plasticity, and neuroendocrine transdifferentiation. In response, novel therapeutic classes—including PROTAC-based AR degraders, N-terminal domain inhibitors, and dual-pathway modulators—have been developed to maintain AR suppression despite complex molecular adaptations [4,200,201,202]. While these approaches are conceptually attractive, their long-term safety, sequencing relative to established agents, and potential for cross-resistance remain incompletely understood, highlighting the need for carefully designed comparative and biomarker-enriched clinical trials.

The integration of radiomics, artificial-intelligence–driven treatment selection, and adaptive therapy principles offers a potential pathway to overcoming many current limitations. AI-enhanced PSMA PET quantification, automated risk-stratification models, and machine-learning algorithms trained on multimodal clinical datasets may improve prediction of response, refine sequencing decisions, and reduce overtreatment [4,203,204,205]. However, these technologies introduce new challenges related to data standardization, interpretability, regulatory oversight, and equitable access, emphasizing that technological sophistication must be matched by pragmatic implementation strategies.

Immunotherapy remains an area of high unmet need in prostate cancer. Although historical efficacy has been modest, emerging approaches combining checkpoint blockade with radioligand-induced immunogenic cell death, PARP inhibitor–mediated STING activation, and modulation of myeloid-driven immune suppression have shown early promise [205,206,207,208]. The heterogeneity of observed responses underscores the importance of personalized immune profiling, yet such approaches are currently resource-intensive and not standardized, limiting near-term applicability. Future research must clarify which immune phenotypes are most likely to benefit and how immunotherapy can be rationally sequenced or combined without excessive toxicity.

Parallel advances in understanding metabolic vulnerabilities—including alterations in cholesterol synthesis, mitochondrial metabolism, oxidative stress pathways, and DNA repair–associated metabolic dependencies—have opened additional therapeutic avenues. Targeting enzymes such as AKR1C3, fatty acid synthase, and regulators of glutamine metabolism has shown potential to sensitize tumors to existing treatments and suppress aggressive phenotypes in resistant disease models [209,210,211]. Translating these findings into clinically viable therapies will require overcoming challenges related to systemic toxicity, metabolic redundancy, and patient selection.

Liquid biopsy technologies are poised to play a central role in addressing many unresolved clinical questions. Serial analysis of circulating tumor cells, ctDNA mutational profiles, and methylation signatures offers a noninvasive means of monitoring tumor evolution, detecting resistance early, and guiding adaptive treatment strategies [212,213,214]. While these approaches promise to reduce unnecessary toxicity and enable more dynamic care, their integration into routine practice will depend on prospective validation, cost containment, and clear demonstration of outcome benefit.

**Figure 8:**
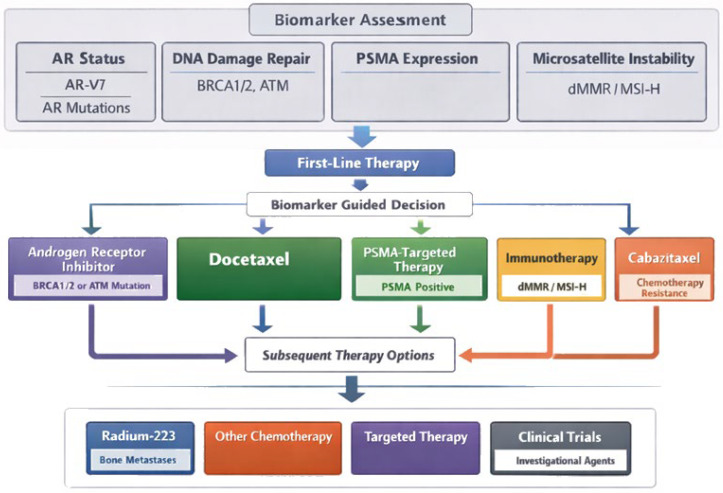
Therapeutic pathways and treatment algorithms in metastatic castration-resistant prostate cancer integrating biomarkers and treatment sequencing, where AR—androgen receptor; AR-V7—androgen receptor splice variant 7; DNA—deoxyribonucleic acid; BRCA1/2—breast cancer gene 1 and 2; ATM—ataxia-telangiectasia mutated; PSMA—prostate-specific membrane antigen; dMMR—deficient mismatch repair; MSI-H—microsatellite instability–high.

Ultimately, the convergence of precision radiotherapy, targeted systemic agents, and advanced molecular diagnostics is likely to define the next phase of prostate cancer management. Increasingly individualized treatment pathways will rely on multidisciplinary collaboration across genetic counseling, molecular pathology, nuclear medicine, urologic oncology, medical oncology, and radiation oncology. This evolution reflects a broader transition toward biology-guided medicine, in which therapeutic decisions are informed less by conventional staging and more by real-time molecular, functional, and imaging biomarkers [215,216,217]. Future research must therefore prioritize not only therapeutic innovation but also implementation science, health equity, and long-term survivorship considerations to ensure that advances in precision oncology translate into meaningful, durable benefit for patients. Table 7 presents contemporary management strategies for prostate cancer, outlining current treatment approaches, while Figure 8 illustrates therapeutic pathways and treatment algorithms in mCRPC, integrating biomarkers and treatment sequencing.

## 6. Conclusions

Emerging therapeutic strategies in prostate cancer reflect a rapidly evolving landscape in which molecular profiling, precision radioligand targeting, and next-generation hormonal manipulation are redefining standards of care. The integration of PARP inhibition with agents such as olaparib, PSMA-targeted radioligand therapy using lutetium (^177^Lu) vipivotide tetraxetan, and potent androgen-receptor pathway suppression with abiraterone has demonstrated that maximal therapeutic benefit is achieved when complementary biological vulnerabilities are addressed concurrently. Together, these modalities underscore a transition from uniform treatment paradigms toward individualized, biology-driven algorithms guided by genomic alterations, functional imaging, and tumor-specific characteristics. Across multiple clinical investigations, both combination and sequential strategies have yielded meaningful improvements in rPFS, biochemical response rates, and OS while maintaining acceptable safety profiles. Despite these advances, significant challenges persist, including the development of acquired resistance, the need for more precise and predictive biomarkers of response, and unresolved questions regarding optimal treatment sequencing to prolong disease control. Future research will increasingly prioritize rational combination strategies, earlier implementation of targeted therapies in hormone-sensitive disease, and refinement of theranostic approaches that integrate molecular imaging with selective delivery of therapeutic payloads. As the evidence base continues to expand, these innovations are expected to converge into comprehensive, biology-driven treatment frameworks that broaden patient eligibility, enhance the depth and durability of response, and ultimately improve long-term outcomes for individuals with advanced prostate cancer.

## Figures and Tables

**Table 1:** TEAEs associated with olaparib and recommended management strategies according to [72,73,74,75,76,77,78,79,80], where TEAE—treatment-emergent adverse event; CBC—complete blood count; 5-HT_3_—5-hydroxytryptamine type 3 (serotonin) receptor; MDS—myelodysplastic syndrome; AML—acute myeloid leukemia; PARP—poly(ADP-ribose) polymerase.

TEAE	Frequency/Severity	Timing/Clinical Features	Recommended Management
Anemia/Hematologic Toxicities	One of the most frequent toxicities; anemia in ~20–30%; grade ≥3 events reported.	Often cumulative; related to bone marrow reserve and prior therapies.	CBC monitoring; dose interruption or reduction; transfusion if indicated; consider growth factors for neutropenia.
Fatigue/Asthenia	Very common; affects ~40% or more of patients; mostly grade 1–2.	Early or cumulative; multifactorial (disease burden, anemia, prior therapy).	Rest, energy pacing, nutrition optimization; psychosocial support; dose reduction if functionally limiting.
Nausea/Vomiting	Very common; nausea > 60%; usually low grade.	Early onset; may persist during treatment.	Antiemetics (5-HT_3_ antagonists, dopamine antagonists); hydration; small frequent meals; interrupt therapy if persistent.
Diarrhea/Dyspepsia/Abdominal Symptoms	Common; typically grade 1–2.	Variable onset; may affect adherence.	Symptomatic treatment (e.g., loperamide); hydration; diet modification; dose reduction if persistent.
Decreased Appetite/Dysgeusia	Common; usually mild.	Gradual onset; may contribute to fatigue and weight loss.	Nutritional counseling; appetite support measures; flavor modification.
Headache/Dizziness	Very common; mostly mild.	Early onset; intermittent.	Analgesics; hydration; evaluate alternative causes if persistent.
Renal Laboratory Changes	Frequent mild creatinine increase; usually low grade.	Early; related to transporter inhibition rather than true renal failure.	Monitor renal function; evaluate progressive or clinically significant elevations.
Hepatic Enzyme Elevation	Uncommon; mostly mild and reversible; rare grade ≥3.	Typically within early months; often asymptomatic.	Periodic liver function tests; dose adjustment or interruption for grade ≥3 toxicity.
Musculoskeletal or Back Pain	Common; mild-to-moderate severity.	Variable; may be related to disease or treatment.	Analgesics; stretching or physiotherapy; dose modification if severe.
Cough/Dyspnea	Uncommon; usually mild.	New or worsening respiratory symptoms; consider pneumonitis if progressive.	Symptomatic care; imaging and pulmonary evaluation if suspected.
Pneumonitis (Serious, rare)	Rare (<1%); potentially life-threatening.	Variable onset; progressive respiratory symptoms.	Immediate treatment interruption; pulmonary consultation; permanent discontinuation if confirmed.
MDS/AML (Rare but serious)	Rare (<1–2%); serious late toxicity.	Late onset; associated with long-term PARP inhibitor exposure.	Long-term CBC monitoring; permanent discontinuation if suspected; hematology referral.

**Table 2:** Major pivotal clinical trials of olaparib, where mCRPC—metastatic castration-resistant prostate cancer; HRR—homologous recombination repair; HRR+/HRR−—HRR positive/negative; BRCA1/2—breast cancer gene 1 and 2; AR—androgen receptor; PFS—progression-free survival; rPFS—radiographic progression-free survival; OS—overall survival; HR—hazard ratio; BICR—blinded independent central review.

Trial	Population/Setting	Design	Key Findings	Key Efficacy Outcomes (Method Specified)
TOPARP-A [42]	mCRPC, biomarker-unselected	Phase II, single-arm	Clinical responses were enriched in patients with HRR gene alterations, establishing the clinical relevance of biomarker stratification.	No comparative hazard ratio (single-arm). Radiologic PFS (investigator-assessed): HRR+ 9.8 months vs. HRR− 2.7 months. OS: HRR+ 13.8 months vs. HRR− 7.5 months.
TOPARP-B [13]	mCRPC with predefined HRR gene mutations	Phase II, randomized (dose comparison), open-label	Higher response rates observed in BRCA1/2-altered tumors compared with other HRR alterations, suggesting differential sensitivity within HRR subgroups.	No standard-of-care comparator; no HR vs. control. Radiologic PFS (investigator-assessed): 5.5 months (olaparib 400 mg) vs. 5.6 months (300 mg). OS: 14.3 months vs. 10.1 months, respectively.
PROfound [12]	mCRPC with HRR gene mutations after progression on AR-targeted therapy	Phase III, randomized, open-label	Olaparib significantly improved radiographic PFS and OS compared with enzalutamide or abiraterone in HRR-mutated mCRPC.	rPFS (BICR, Cohort A): HR 0.34; median 7.4 vs. 3.6 months. OS (final analysis, Cohort A): HR 0.69; median 19.1 vs. 14.7 months.
PROpel [56]	First-line mCRPC, all-comers	Phase III, randomized, double-blind	Olaparib plus abiraterone improved radiographic PFS compared with abiraterone alone, with the greatest magnitude of benefit observed in HRR-mutated tumors.	rPFS (investigator-assessed, primary): HR 0.66; median 24.8 vs. 16.6 months. OS (final prespecified analysis): HR 0.81; median 42.1 vs. 34.7 months (did not meet prespecified significance threshold).
MAGNITUDE [81]	First-line mCRPC stratified by HRR mutation status	Phase III, randomized, double-blind	Niraparib plus abiraterone improved outcomes in HRR-mutated disease, while no benefit was observed in HRR-negative patients, leading to early closure of that cohort.	rPFS (BICR): BRCA1/2 subgroup HR 0.53; median 16.6 vs. 10.9 months. Overall HRR+ HR 0.73; median 16.5 vs. 13.7 months. OS (final): HRR+ HR 0.93 (no OS benefit); BRCA1/2 OS HR 0.79; median 30.4 vs. 28.6 months.

**Table 3:** TEAEs associated with lutetium (^177^Lu) vipivotide tetraxetan and recommended management strategies according to [125,126,127,128,129,130,131], where TEAE—treatment-emergent adverse event; CBC—complete blood count; G-CSF—granulocyte colony-stimulating factor; LFT—liver function test; MDS—myelodysplastic syndrome; AML—acute myeloid leukemia.

TEAE	Frequency/Severity	Timing/Clinical Features	Recommended Management
Fatigue	Very common; mostly grade 1–2; occasional grade ≥3.	Cumulative; multifactorial (disease burden, anemia, prior therapies).	Evaluate reversible causes; encourage activity/exercise as tolerated; treat anemia; delay therapy for grade ≥3.
Xerostomia/Salivary Gland Toxicity	Very common (~30–60%); predominantly grade 1–2.	Early onset; may persist; impacts taste, oral comfort, and dental health.	Oral hygiene measures; dental review; sialagogues, saliva substitutes, hydration; investigational cooling strategies where available.
Nausea/Vomiting	Common; usually low grade.	Peri-treatment or delayed onset.	Antiemetics; hydration; small frequent meals; evaluate alternative causes if persistent.
Decreased Appetite/Weight Loss/Constipation	Common; generally low–to-moderate severity.	Multifactorial; often overlaps with fatigue and GI symptoms.	Nutritional support; antiemetics as appropriate; appetite support; bowel regimen.
Hematologic Toxicities (Anemia, Thrombocytopenia, Neutropenia)	Very common as laboratory abnormalities; grade ≥3 events observed.	Delayed nadir (≈4–8+ weeks); cumulative risk, higher with extensive bone or marrow involvement.	Regular CBC monitoring; hold or delay dosing per label; transfusions as indicated; G-CSF per guidelines; long-term surveillance.
Renal Toxicity (Creatinine Increase)	Uncommon.	Risk increased with baseline renal impairment or dehydration.	Ensure hydration; avoid nephrotoxins; monitor renal function; interrupt or delay therapy if clinically indicated.
Hepatic Enzyme Elevation	Uncommon; usually mild and transient.	Often asymptomatic; detected on routine labs.	Periodic LFT monitoring; interrupt for clinically significant elevations; modify or discontinue if persistent.
Pulmonary Events (Pneumonitis)	Rare; isolated cases reported.	New or worsening respiratory symptoms.	Prompt imaging and infection work-up; corticosteroids if inflammatory etiology suspected; discontinue therapy if severe or confirmed.
Secondary Malignancies (MDS/AML)	Rare; observed during long-term follow-up.	Late onset following cumulative exposure.	Long-term CBC monitoring; hematology referral if suspected; patient counseling regarding long-term risk.
Other (Infusion Reactions, Alopecia, Minor Laboratory Abnormalities)	Mostly low grade.	Variable timing.	Standard supportive care and routine monitoring.

**Table 4:** Major pivotal clinical trials of lutetium (^177^Lu) vipivotide tetraxetan, where mCRPC—metastatic castration-resistant prostate cancer; PSMA—prostate-specific membrane antigen; ARPI—androgen receptor pathway inhibitor; SOC—standard of care; rPFS—radiographic progression-free survival; OS—overall survival; BICR—blinded independent central review; HR—hazard ratio; PSA50—≥50% decline in prostate-specific antigen; PFS—progression-free survival; FDG—fluorodeoxyglucose; PET—positron emission tomography; ADT—androgen deprivation therapy; mHSPC—metastatic hormone-sensitive prostate cancer.

Trial	Population/Setting	Design	Key Findings	Key Efficacy Outcomes (Method Specified)
VISION (Phase III) [21,126,131]	mCRPC, PSMA-positive, previously treated with ARPI and 1–2 taxanes	Phase III, open-label, randomized (2:1): ^177^Lu-PSMA-617 + SOC vs. SOC	Met primary endpoints with significant improvement in OS and rPFS; safety profile manageable with expected hematologic and salivary toxicities.	rPFS (BICR): HR 0.40; median 8.7 vs. 3.4 months. OS (final): HR 0.62; median 15.3 vs. 11.3 months.
TheraP (Phase II) [22,129]	mCRPC, cabazitaxel-eligible, PSMA-positive (dual PSMA/FDG PET selection)	Phase II, randomized, open-label: ^177^Lu-PSMA-617 vs. cabazitaxel	Higher PSA50 response rates and more favorable toxicity profile with ^177^Lu-PSMA-617 compared with cabazitaxel.	PFS (investigator-assessed): HR ~0.63; median 5.1 vs. 3.4 months. OS: immature at primary analysis; no statistically significant difference reported.
PSMAfore (Phase III) [132]	Taxane-naïve mCRPC, progressed after one ARPI, PSMA-positive	Phase III, randomized, open-label: ^177^Lu-PSMA-617 vs. change of ARPI	Significant improvement in rPFS; improvements in health-related quality of life and pain outcomes; OS data immature with crossover allowed.	rPFS (BICR, interim): HR 0.41; median ~12.0 vs. ~5.6 months. OS: immature (crossover permitted).
PSMAddition (Phase III) [125]	Metastatic hormone-sensitive prostate cancer (mHSPC), PSMA-positive	Phase III, randomized: ^177^Lu-PSMA-617 + ADT/ARPI vs. ADT/ARPI alone	Interim analysis demonstrates rPFS benefit with addition of radioligand therapy; OS follow-up ongoing.	rPFS (BICR, interim): HR ~0.71; median not reached in either arm. OS: ongoing.
Phase II/Single-Arm Studies [133]	Various mCRPC populations with heterogeneous prior therapies	Prospective single-arm studies; multiple dosing regimens	Demonstrated consistent antitumor activity with PSA responses in approximately 50% of patients and generally favorable tolerability.	No comparative HR (single-arm). Median PFS typically ~4–8 months. Median OS ~12–15 months, depending on cohort and prior therapy.

**Table 5:** TEAEs associated with abiraterone and recommended management strategies according to [163,164,165], where TEAE—treatment-emergent adverse event; LFT—liver function test; ALT—alanine aminotransferase; AST—aspartate aminotransferase; ULN—upper limit of normal; MI—myocardial infarction; GI—gastrointestinal; IV—intravenous.

TEAE	Frequency/Severity	Timing/Clinical Features	Recommended Management Strategies
Hypertension	Very common; predominantly grade 1–2; occasional grade ≥3.	Early onset or cumulative; may exacerbate pre-existing hypertension.	Regular blood pressure monitoring; initiate or optimize antihypertensive therapy; consider temporary interruption for severe or uncontrolled hypertension.
Hypokalemia	Common; grade ≥3 events reported in a subset of patients.	Early, particularly with concomitant corticosteroids or diuretics.	Monitor serum potassium; oral or IV supplementation as needed; review and adjust concomitant medications.
Fluid Retention/Edema	Common; mostly grade 1–2.	Cumulative; peripheral edema most frequent, occasionally generalized.	Monitor weight and clinical signs; sodium restriction; diuretics as indicated; evaluate cardiac function if persistent or progressive.
Hepatotoxicity/LFT Elevation	Common; grade ≥3 elevations less frequent.	Typically within the first few months; often asymptomatic.	Baseline and periodic LFT monitoring; hold or discontinue therapy for ALT/AST >5× ULN; dose modification per label.
Fatigue/Asthenia	Very common (all grades); mostly grade 1–2.	Early or cumulative; multifactorial (disease burden, concomitant therapies).	Assess reversible contributors (anemia, sleep disturbance); energy conservation; moderate activity as tolerated; supportive care.
Cardiac Events (MI, Arrhythmia, Heart Failure–rare)	Uncommon but potentially serious.	More likely in patients with pre-existing cardiovascular disease.	Baseline cardiovascular assessment; monitor for new cardiac symptoms; optimize cardiac medications; interrupt therapy for severe events.
Musculoskeletal Pain/Arthralgia	Common; mostly grade 1–2.	Often early; may persist during treatment.	Analgesics and physiotherapy; assess for progression of metastatic bone disease if symptoms worsen.
Diarrhea/GI Upset/Nausea	Common; usually mild.	Can occur at any time during therapy.	Symptomatic management with antidiarrheals, antiemetics, hydration; dietary modifications as needed.
Mineralocorticoid Excess–Related Symptoms (e.g., Headache, Hypokalemia, Edema)	Common; related to observed hormonal effects in prostate cancer trials.	Early; may coincide with hypertension or electrolyte abnormalities.	Monitor blood pressure and electrolytes; low-dose corticosteroid co-administration per label; treat individual symptoms as above.
Other (Rash, Laboratory Abnormalities–rare)	Low frequency.	Variable timing.	Symptomatic management; interrupt or discontinue therapy if severe; routine laboratory monitoring.

**Table 6:** Major pivotal clinical trials of abiraterone, where mCRPC—metastatic castration-resistant prostate cancer; rPFS—radiographic progression-free survival; OS—overall survival; HR—hazard ratio; ADT—androgen deprivation therapy; mHSPC—metastatic hormone-sensitive prostate cancer.

Trial	Population/Setting	Design	Key Findings	Key Efficacy Outcomes (Method Specified)
Phase II/Single-Arm Studies [180]	mCRPC across various prior therapy exposures	Prospective, single-arm	Demonstrated clinical activity and tolerability of abiraterone in both pre- and post-chemotherapy settings, supporting further randomized evaluation.	No comparative HR (single-arm). Median rPFS ~3–6 months. Median OS ~12–15 months, depending on prior therapy exposure.
COU-AA-301 (Phase III) [26,27]	mCRPC post-docetaxel	Phase III, randomized, double-blind: abiraterone + prednisone vs. placebo + prednisone	Abiraterone significantly improved OS and rPFS with a manageable safety profile in post-chemotherapy mCRPC.	rPFS (investigator-assessed): HR ~0.67; median 5.6 vs. 3.6 months. OS: HR 0.65; median 14.8 vs. 10.9 months.
COU-AA-302 (Phase III) [28,142]	Chemotherapy-naïve mCRPC	Phase III, randomized, double-blind: abiraterone + prednisone vs. placebo + prednisone	Abiraterone improved OS, delayed radiographic progression, and maintained quality of life in chemotherapy-naïve mCRPC.	rPFS (investigator-assessed): HR 0.43; median 16.5 vs. 8.3 months. OS (final): HR 0.81; median 34.7 vs. 30.3 months.
LATITUDE (Phase III) [29,143]	Newly diagnosed high-risk metastatic hormone-sensitive prostate cancer (mHSPC)	Phase III, randomized, double-blind: abiraterone + ADT vs. placebo + ADT	Addition of abiraterone to ADT significantly improved OS and rPFS in high-risk mHSPC.	rPFS (investigator-assessed): HR 0.47; median 33.0 vs. 14.8 months. OS (final): HR 0.62; median 53.3 vs. 36.5 months.
STAMPEDE (Arm G, Phase III) [181,182]	Newly diagnosed mHSPC or high-risk locally advanced prostate cancer	Multi-arm, multi-stage, randomized: abiraterone + ADT ± radiotherapy vs. ADT alone	Abiraterone plus ADT improved OS and failure-free survival across metastatic and high-risk non-metastatic populations.	OS: HR 0.63; 3-year OS 83% vs. 76%. rPFS: median not reached at primary reporting. Failure-free survival: HR ~0.29.
CYCLONE 2 (Phase III) [183]	mCRPC	Phase III, randomized, double-blind: abemaciclib + abiraterone vs. placebo + abiraterone	Did not meet the primary rPFS endpoint; no improvement in efficacy with the addition of abemaciclib.	rPFS: HR ~1.0; median rPFS similar between arms. OS: immature; no benefit demonstrated at reporting.

**Table 7:** Contemporary management of prostate cancer: current methods of treatment, where ADT—androgen deprivation therapy; AR—androgen receptor; mCRPC—metastatic castration-resistant prostate cancer; mHSPC—metastatic hormone-sensitive prostate cancer; OS—overall survival; PSA—prostate-specific antigen; rPFS—radiographic progression-free survival; RT—radiation therapy.

Treatment Modality	Indication/Patient Population	Key Notes/Considerations
Active Surveillance	Low-risk localized prostate cancer	Regular PSA monitoring, periodic biopsy or MRI; avoids overtreatment; patient selection critical [218,219]
Radical Prostatectomy	Localized or locally advanced prostate cancer	Open, laparoscopic, or robotic approaches; may be combined with pelvic lymph node dissection; consider functional outcomes (continence, potency) [220,221]
Radiation Therapy	Localized or locally advanced disease; salvage therapy	External beam RT (EBRT) or brachytherapy; can be combined with ADT for intermediate/high-risk disease; fractionation schemes vary [222,223]
Androgen Deprivation Therapy (ADT)	Advanced or metastatic prostate cancer; often combined with radiation	LHRH agonists/antagonists or surgical castration; monitor for metabolic and cardiovascular side effects; can be intermittent or continuous [27,224]
Next-Generation AR Pathway Inhibitors	mCRPC or high-risk hormone-sensitive disease	Abiraterone, enzalutamide, apalutamide, darolutamide; improve OS and rPFS; monitor for hypertension, hepatotoxicity, fatigue [170,225]
Chemotherapy	mCRPC; selected high-risk mHSPC	Docetaxel or cabazitaxel; improves OS in metastatic setting; monitor hematologic toxicity [226,227]
Radioligand Therapy (RLT)	PSMA-positive mCRPC after standard therapies	^177^Lu-PSMA-617; targeted therapy; monitor hematologic, renal, salivary gland toxicities; imaging required for eligibility [21,22]
Immunotherapy	MSI-H/dMMR or select mCRPC	Pembrolizumab or other checkpoint inhibitors; only effective in biomarker-selected populations [228]
Bone-Targeted Therapy	Metastatic disease with bone involvement	Denosumab or zoledronic acid; radium-223 for symptomatic bone metastases; prevents skeletal-related events [229,230]
Multimodal/Combination Therapy	High-risk localized or metastatic disease	Combining ADT, RT, surgery, and systemic therapy as appropriate; individualization based on risk, comorbidities, and molecular features [231,232,233,234]

## Data Availability

No new data were created or analyzed in this study. Data sharing is not applicable to this article.

## Abbreviations

The following abbreviations are used in this manuscript:

^177^Lu-PSMA-617	Lutetium-177–Labeled PSMA-617 Radioligand
ADT	Androgen Deprivation Therapy
ALT	Alanine Aminotransferase
AML	Acute Myeloid Leukemia
AR	Androgen Receptor
ARPI	Androgen Receptor Pathway Inhibitor
ARSI	Androgen Receptor Signaling Inhibitor
ARV	Androgen Receptor Variants
AR-V7	Androgen Receptor Splice Variant 7
AST	Aspartate Aminotransferase
ATM	Ataxia-Telangiectasia Mutated
BICR	Blinded Independent Central Review
BP	Blood Pressure
BRCA	Breast Cancer Susceptibility Gene
BRCA1/2	Breast Cancer Susceptibility Gene 1 or 2
CBC	Complete Blood Count
ctDNA	Circulating Tumor DNA
CYP17A1	Cytochrome P450 17A1
dMMR	Deficient Mismatch Repair
DHT	Dihydrotestosterone
DNA	Deoxyribonucleic Acid
FDG	Fluorodeoxyglucose
FDG PET	^18^F-Fluorodeoxyglucose Positron Emission Tomography
G-CSF	Granulocyte Colony-Stimulating Factor
GI	Gastrointestinal
HR	Hazard Ratio
HRD	Homologous Recombination Deficiency
HRQOL	Health-Related Quality of Life
HRR	Homologous Recombination Repair
HRR+/HRR−	Homologous Recombination Repair Positive/Negative
HSD3B	3β-Hydroxysteroid Dehydrogenase
IV	Intravenous
LFT	Liver Function Test
mCRPC	Metastatic Castration-Resistant Prostate Cancer
mCSPC	Metastatic Castration-Sensitive Prostate Cancer
mHSPC	Metastatic Hormone-Sensitive Prostate Cancer
MDS	Myelodysplastic Syndrome
MI	Myocardial Infarction
MSI-H	Microsatellite Instability–High
NR	Not Reached
OS	Overall Survival
P	Phosphate/Phosphorylation
PARP	Poly(ADP-Ribose) Polymerase
PET	Positron Emission Tomography
PFS	Progression-Free Survival
PSA	Prostate-Specific Antigen
PSA50	≥50% Decline in Prostate-Specific Antigen
PSMA	Prostate-Specific Membrane Antigen
QoL/QOL	Quality of Life
rPFS	Radiographic Progression-Free Survival
RLT	Radioligand Therapy
RT	Radiation Therapy
SMPC	Summary of Product Characteristics
SOC	Standard of Care
SRD5A	Steroid 5-Alpha-Reductase
TEAE	Treatment-Emergent Adverse Event
ULN	Upper Limit of Normal
